# CoCross: An ICT Platform Enabling Monitoring Recording and Fusion of Clinical Information Chest Sounds and Imaging of COVID-19 ICU Patients

**DOI:** 10.3390/healthcare10020276

**Published:** 2022-01-30

**Authors:** Vassilis Kilintzis, Nikolaos Beredimas, Evangelos Kaimakamis, Leandros Stefanopoulos, Evangelos Chatzis, Edison Jahaj, Militsa Bitzani, Anastasia Kotanidou, Aggelos K. Katsaggelos, Nicos Maglaveras

**Affiliations:** 1 Laboratory of Computing, Medical Informatics and Biomedical—Imaging Technologies, The Medical School, Aristotle University of Thessaloniki, 54124 Thessaloniki, Greece; billyk@auth.gr (V.K.); beredim@auth.gr (N.B.); lstefano@auth.gr (L.S.); chatzise@auth.gr (E.C.); 2 1st Intensive Care Unit, “G. Papanikolaou” General Hospital of Thessaloniki, 57010 Pilea Hortiatis, Greece; vakaimak@gmail.com (E.K.); bitmilly@gmail.com (M.B.); 3 First Department of Critical Care Medicine and Pulmonary Services, Evangelismos Hospital, The Medical School, National and Kapodistrian University of Athens, 10676 Athens, Greece; edison.jahaj@gmail.com (E.J.); akotanid@uoa.gr (A.K.); 4 Department of Electrical and Computer Engineering, Northwestern University, Evanston, IL 60201, USA; a-katsaggelos@northwestern.edu

**Keywords:** biomedical monitoring, COVID-19, ICU, ICT platform, lung sounds, heart sounds, X-rays, lung ultrasound, research database

## Abstract

Monitoring and treatment of severely ill COVID-19 patients in the ICU poses many challenges. The effort to understand the pathophysiology and progress of the disease requires high-quality annotated multi-parameter databases. We present CoCross, a platform that enables the monitoring and fusion of clinical information from in-ICU COVID-19 patients into an annotated database. CoCross consists of three components: (1) The CoCross4Pros native android application, a modular application, managing the interaction with portable medical devices, (2) the cloud-based data management services built-upon HL7 FHIR and ontologies, (3) the web-based application for intensivists, providing real-time review and analytics of the acquired measurements and auscultations. The platform has been successfully deployed since June 2020 in two ICUs in Greece resulting in a dynamic unified annotated database integrating clinical information with chest sounds and diagnostic imaging. Until today multisource data from 176 ICU patients were acquired and imported in the CoCross database, corresponding to a five-day average monitoring period including a dataset with 3477 distinct auscultations. The platform is well accepted and positively rated by the users regarding the overall experience.

## 1. Introduction

In the media briefing on March 11, 2020, the World Health Organization (WHO) declared a state of a pandemic for COVID-19. Patients with a SARS-CoV-2 infection can develop coronavirus disease 2019 (COVID-19) [1] with clinical manifestations ranging from mild respiratory symptoms to severe pneumonia and a fatality rate estimated at around 2%. The first case of the coronavirus disease 2019 (COVID-19) was reported in Wuhan, Hubei province, China in December 2019 [2]. The fast spread at least at the beginning of this pandemic outbreak is attributed to the lengthy incubation period ranging from 2 to 14 days, and the modern way of living, where fast transportation and traveling are major characteristics. The effects of COVID-19 were devastating in countries where the infection progressed for a full month before the world realized the severity and the health hazard scale of COVID-19 [3]. COVID-19 has caused a large number of cases with respiratory problems in the form of pulmonary infiltrates, progressive worsening dyspnea and respiratory failure [4]. In many cases, patients develop a serious illness that requires intubation and hospitalization in an intensive care unit (ICU) for mechanical ventilation [5]. Severely ill patients with a COVID-19 infection show frequent alternations between different clinical and radiological patterns [6], as well as frequent complications of the disease and applied mechanical ventilation (such as atelectasis, pneumothorax, pulmonary embolism, etc.). In many cases, the disease is complicated by acute myocardial injury often affecting the cardiac valves. All the above conditions should be diagnosed fast to be treated appropriately. The treatment of severely ill patients with the COVID-19 virus poses many challenges due to the multi-organ deficiencies caused by the virus and thus it requires constant monitoring of the parameters and imaging primarily of the respiratory and cardiovascular systems. At the same time, traditional monitoring methods are difficult to apply within the context of personal protective equipment used by the medical staff. The most important of these methods is the auscultation of the respiratory system and heart sounds with the use of a stethoscope. The auscultation technique has remained critical in the comprehensive diagnosis and management of the COVID-19 disease, despite the practical issues related to the personal protective equipment [7], since this method is easy to apply multiple times a day, reliable, and intensivists are highly familiar with it. In addition, modern digital auscultation modalities have been reported to change the landscape of diagnosis and treatment of many heart and lung conditions in a favorable way for the patients [8]. Due to the inability to apply heart-lung auscultations, the current scientific reports on COVID-19 virus infection do not refer to auditory findings from the disease [5,9]. Intensive care physicians are required to resort to alternative testing methods (e.g., the application of ultrasound) that lack the immediacy and practicality of listening with the stethoscope and cannot substitute the information gained from the auscultation method. At the same time, even diagnostic modalities that are usually quick and easy, like the bedside chest X-ray, are burdensome to execute and require more extensive planning, due to the need for a dedicated X-ray machine on a constant basis in the infected ICU area and the time-consuming donning and doffing of the personal protective equipment by the team of radiologists. Furthermore, CT scans are often impossible to have, in the most severe cases of ARDS patients (acute respiratory distress syndrome) who are unable to be transported safely to the CT scanner and back.

One of the biggest issues in treating COVID-19 positive subjects is how to achieve early detection of the symptoms that indicate deterioration of the disease status and requirement for hospitalization and/or early treatment interventions [10]. Such symptoms may include alterations in heart rate, oxygen saturation of hemoglobin (SpO2), elevated temperature and cough episodes. Moreover, auditory findings in chest auscultations may reveal early signs of acute onset of complications during the management of severely ill COVID-19 patients (examples include pneumothorax/barotrauma [11], new ventilator-associated pneumonia, atelectasis and progression to pulmonary fibrosis in case of newly identified presence of squawks [12]).

It is evident that there is a need for an easily accessible and simple-to-use set of devices that could provide this assessment in an effective and continuous manner. This applies to both people at the community level with the need for remote monitoring (e.g., families of patients that recovered from COVID-19) and healthcare professionals dealing with the COVID-19 pandemic in the ICU or other clinical settings. Additionally, databases containing auscultation data from ICUs from patients with COVID-19 can help better understand the lung and heart pathophysiology changes due to the disease [13]. The integration of heart and lung sounds with the biosignals and bioparameters recorded in the ICU and their analysis, such as, HRV, temperature entropy, respiratory signals’ features, etc. could improve identification or prediction of the disease progress, leading to possible life saving interventions in COVID-19 patients.

Therefore, there is a need to implement an alternative method of bedside monitoring to patients with COVID-19 who are treated in the ICU, ideally combining different diagnostic techniques and transmitting the recordings wirelessly as fast as possible for further assessment by the ICU team of physicians. There is also a certain need for efficient and reliable bedside diagnostic and imaging modalities that also give insight to respiratory dynamics and cardiorespiratory interactions, to enable intensivists to evaluate the effect of the ventilator settings on the cardiorespiratory dynamics, regardless of the underlying cause for the patient’s critical condition and the need for mechanical ventilation [10].

The CoCross Platform is a complete system for monitoring the evolution of lung and heart sounds and diagnostic imaging of patients with COVID-19 who are treated in the ICU, enhancing collaboration of experts, and overcoming the difficulties that are imposed by the a) use of protective equipment and b) restrictions on hardware transfer in-and-out of the ICU due to COVID-19. The CoCross Platform consists of the following components, (1) the CoCross services back-end, hosting, the authorization and authentication service, the data management service, and the web application for intensivists, (2) the CoCross tablet hosting the CoCross4Pros application along with the device-specific add-on modules for monitoring auscultation and vital signs, and (3) the commercially available, medical devices for the acquisition of auscultation and vital signs. It enables almost real-time hearing of annotated, with regards to the location of the auscultation, lung and heart sounds outside the ICU along with accompanying data such as contextual information (e.g., ventilator parameters, clinical conditions) and oximeter measurements. The data are securely stored and can be easily shared among intensivists retaining authorization and access control measures. The CoCross platform is secure, lightweight, and scalable and provides isolated control of data per clinic/ICU while also being easily expansible to support additional biomedical devices for the acquisition of additional vital signs if needed, without rebuilding/updating the CoCross4Pros application. Biomedical devices can be a spirometer, a scale, a glucose monitor, a blood pressure monitor or an ECG monitor. Data acquired using CoCross are used for building a new multisource annotated database that would enable the development of advanced digital diagnostics for COVID-19 patients.

## 2. State of the Art

Given the severity and persistence of the COVID-19 infection, which is manifested in heavy lung dysfunction causing dyspnea and increased respiration ineffective efforts due to fibrosis, as well as failure in the heart mechanical functionality and kidney dysfunction, there is an urgent need for the development of eHealth systems for use in the clinical environment, as well as outside the hospital for a continuous monitoring of the evolution of the COVID-19 infection and its comorbidities for the optimal therapeutic and preventive health services to the citizens of the world. Some countries such as South Korea, have already developed new ICT systems that are embedded in the hospital information systems (HIS) to tackle the unique problems introduced from the COVID-19 epidemic [14]. In this work a dashboard was created for the medical staff to view the vital signs and symptoms of all patients via a mobile app used by the patients. Cloud-based image sharing enabled interoperability between the country’s medical institutions. This approach “flattened the curve” of the rate of infection in South Korea. It is evident that inter/intra-hospital systems containing COVID-19 information and data are catalysts for reducing the COVID-19 patients and managing the COVID-19 patients after the infection.

The South Korea paradigm, as well as the China paradigm, show that the COVID-19 pandemic has proved the need for upscaling and accelerating the industrial information integration (III) process, for the health care system and society to tackle worldwide pandemics and acute healthcare needs [15]. It is true that in the ICT field, progress is not harmonized, and this creates implementation issues at the country, continent, and world scale due to the problems in the information integration networks (IIN).

The availability of a wealth of wearable sensors, as well as advanced technologies for monitoring of biosignals, bioparameters and lung imaging, along with a new generation of ICU equipment such as ventilators with EIT (electrical impedance tomography) capacity are providing new tools and measurements (e.g., thoracic bio-impedance measurements) which can shed light in the elucidation of the COVID-19 induced mechanisms and thus contribute in the advancement of lung dysfunction digital diagnostics [16]. The need for big data related to lung function such as lung sounds, respiratory rate estimation, heart sounds, to name a few, are primary biosignals used in the assessment of the lung function, and on the classification of COVID-19/non-COVID-19 patients, as well as on helping with the prediction of the evolution of the disease in ICU COVID-19 patients. For example, in 2020 in the USA the National Institutes of Health launched the National COVID Cohort Collaborative to build a centralized national data resource.

Additionally, a number of COVID-19 mHealth systems have been developed for recording in EHR and PHR anamnesis data, as well as public health-related data for patients who were tested positive with the COVID-19 virus either being at home or in the hospital (COVID-19 clinics, ICU) [17]. There is a trend for the development of complementary approaches to enable industrial information integration and industrial innovation to tackle the COVID-19 pandemic although there are still gaps [18].

From the experience until now, about 18 months after the breakout of the COVID-19 pandemic, most of the effort at least in the beginning concentrated on developing methodologies and systems to classify COVID vs non-COVID subjects, for use both in the hospitals, as well as in the general population. A number of advanced technologies from nanomedicine to artificial intelligence (AI) to cloud computing to Medical Informatics to tissue engineering and more are being developed to tackle specific problems dealing with COVID-19 such as prevention, cure, disease management, resource optimization and of course data analytics and biomedical information optimal use [19]. For example, medical imaging technologies are at the forefront of combating COVID-19. Especially the use of X-rays and CTs for COVID patients’ classification attracted a lot of interest, due to the availability of X-rays and CTs in all tiers of the healthcare system. Several AI techniques have been proposed, mostly based on AI/DL approaches, with excellent results even in multinational datasets [20,21]. 

One of the current gaps in dealing with COVID-19 is the lack of a widely accepted protocol in dealing with COVID-19 patients in COVID clinics and in ICUs. The problem is the difficulty in assessing the pathophysiology of the lungs and cardiovascular system dynamics, especially in the presence of comorbidities and aging, which in many cases can lead to worsening of the patient’s condition or death. Thus, the unavailability of annotated databases from ICUs with recordings of biosignals, bioparameters and bioimages related to the functionality of the lungs and the cardiovascular system at large, as well as the relationship of these recording and data with the ICU standard recorded parameters and scores (e.g., APACHE, SOFA) renders the monitoring, medical decision making and interventions a difficult to tackle the problem in COVID-19 ICUs. The most complete existing open-access databases with some annotations and patient information are the MIMIC II [22] and MIMIC III [23] databases that can be found in PHYSIONET, which have contributed largely to the development of predictive models, diagnostics and testing of AI/ML approaches in ICU data, related to cardiac arrest [24], respiratory complications, septic shock, and outcome.

The aims of CoCross are (1) to provide a user friendly and easy to deploy solution for lung and heart sound monitoring of COVID-19 patients receiving care in an ICU, (2) expand the knowledge gained with the development of the ICBHI respiratory database [25] with the annotated adventitious lung sounds for different lung pathologies, with a new annotated database of data from the ICU, (3) propose an interoperable and expandable platform for integrating multisource information and data that would enable the development of advanced digital diagnostics for COVID-19 patients and accelerate the industrial information integration (III) process in tackling the COVID-19 pandemic [15].

## 3. Materials and Methods

The CoCross platform, in terms of software, is deployed in two locations; the first is the server (CoCross back-end) hosting, in distinct virtualized containers, the CoCross services and the second is the CoCross tablet hosting the CoCross4Pros application for healthcare professionals along with the applications/modules that are specific to each medical device. 

**Figure 1:**
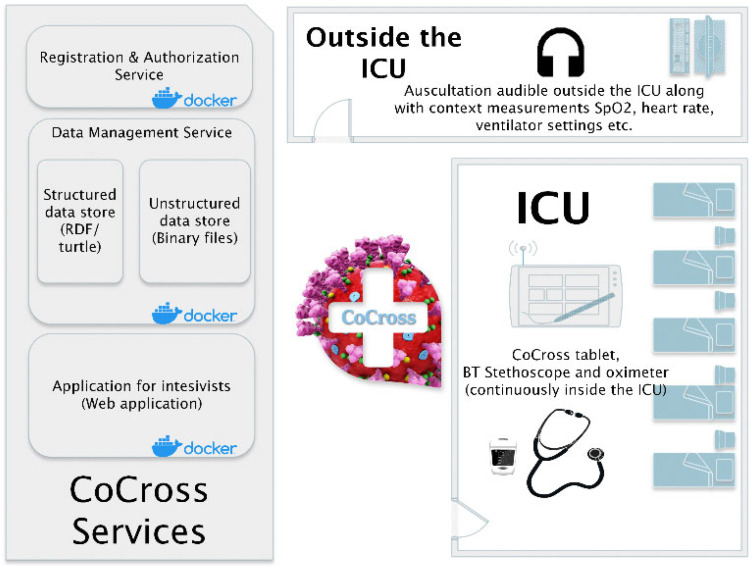
The CoCross platform.

The CoCross platform is depicted in Figure 1. The ICU corresponds to the location where the COVID-19 patients are treated, isolated from the rest of the clinic with equipment that is either disposable or they do not exit the location once they are used inside. Due to those restrictions the devices, the tablet, the pulse oximeter, the ultrasound probe and the stethoscope never exit the ICU. These restrictions apply also to simpler objects such as paper notes that may not exit the ICU as well, thus, making it difficult to transfer patient-related information from the intensivists inside the ICU to those outside.

In each tablet inside the ICU, the CoCross4Pros application is installed along with distinct applications for each wirelessly connected device (i.e., pulse oximeter and stethoscope). The CoCross4Pros application is managing user authentication, the selection of specific patients, the initialization and termination of a recording session, and the registration of in-ICU notes for the specific patient by the intensivist in charge. It also manages the manual entry of in-ICU accessible values, such as ventilator parameters (e.g., ventilator mode, positive end exhalation pressure (PEEP), etc.), as well as uploading of DICOM files captured by the in-ICU stationed portable ultrasound device. The per-device module applications manage the connectivity with each specific device, setting-up specific parameters either for the device itself or meta information for the specific measurement/signal acquisition.

Outside the ICU, in one or more terminals, using the cross-browser, a web-based application for intensivists, the authorized professionals may access all information that was acquired in the recording sessions performed on a specific patient inside the ICU. They can listen to the auscultation sounds that were recorded only a few seconds ago (40–120 s in normal operation) inside the ICU without the burden of the protective equipment. Using the application for intensivists, the professionals also have access to automatically uploaded ultrasound acquisitions along with patient demographics information and relevant health data.

The CoCross back-end hosts the three components that support the CoCross platform. The authentication and authorization service is based on Keycloak (www.keycloak.org accessed on 20 April 2021), which is a widely accepted open-source identity and access management solution and manages user access on both the application for intensivists and the CoCross4Pros mobile application and the sharing procedure of patient data. The data management service is based on the in-house developed-flexible data management framework for integrated care [26] that leverages HL7 FHIR ontologies to provide storing and conditional retrieval of all the CoCross data using RDF/turtle or JSON-LD as a data exchange format. The third component hosted on the CoCross back-end is the web application for intensivists, developed on top of the Laravel framework (www.laravel.com accessed on 20 April 2021) and the Model/View/Controller MVC) software design pattern that separates internal representations of information from the way information is presented to the user. The application for intensivists unifies the information retrieved in all the recording sessions of a specific patient and properly presents the data (DICOM image series, auscultation sounds, oximetry measurements, ventilator parameters, etc.) with an easy-to-use graphical interface accessible from PCs and tablet devices. 

In the next section, the architecture and deployment decisions of the CoCross services and CoCross mobile applications are presented.

### 3.1. The CoCross Architecture

**Figure 2:**
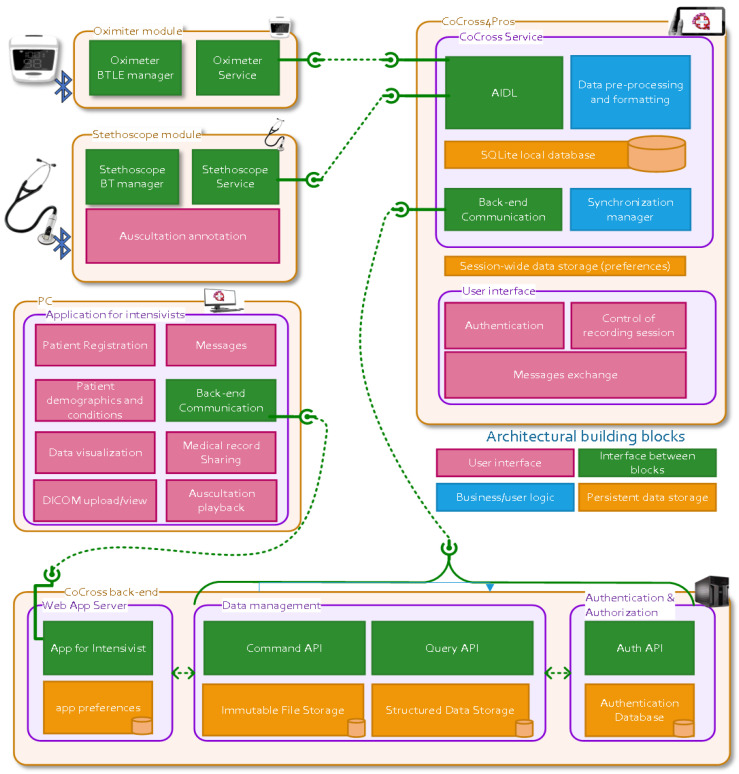
The CoCross conceptual architecture diagram.

In Figure 2 the diagram of the CoCross architecture is presented. The displayed components are the CoCross backend, the CoCross4Pros tablet-based application, the web application for intensivists and the device specific modules for acquiring data from the oximeter and the stethoscope. In the diagram for each component the architectural building blocks, color-coded by their functionality (i.e., user interface, interconnection, business logic/processing and storage) are also presented. 

CoCross is built on linked data [27], HL7 FHIR (www.hl7.org/fhir accessed on 20 April 2021) and ontologies. The semantic data model, defined as an OWL ontology, based on HL7 FHIR resources and datatypes [28], defines the semantics of the data that are managed in the context of CoCross. The flexible data management framework [26] leverages those semantics to provide a RESTful web service API to be used by the client applications (i.e., the tablet based CoCross4Pros and the web application for intensivists). The client applications use the ontology first, as a reference of the concepts that are managed, along with their specific semantics, such as accepted data types, cardinality restrictions or concept grouping (e.g., SpO2 value is accepted as exactly one simple quantity and is a subclass of VitalSigns class) and, secondly, to define the format of the exchanged data since class instances correspond to the actual payload of requests and responses of the API. 

### 3.2. The CoCross Services

The CoCross services’ implementation represents the latest iteration of our in-house developed data management framework for health data. Compared to previously published work [26]:

- Data creation and data retrieval API endpoints and implementations have been separately packaged. This command query separation allows us to scale those two different aspects of data management independently, depending on the deployment scenario’s requirements.
- Authentication and authorization services are now available allowing us to define and enforce authorization access rules for specific data resources.
- Each service component is now packaged as a separate Docker container, and supports horizontal scaling, allowing our framework to be deployed to a wide variety of on-premises or cloud scenarios.

The CoCross services are:

- The authentication and authorization services, using Keycloak and its OpenID Connect implementation. These services allow us to manage user registration, patient registration and data sharing.
- The data management services are built and deployed using standard Java EE technologies. Data management primarily refers to creating and querying for health data, including unstructured data such as auscultation recordings. In addition, the system’s data model is also managed via a service, and its development is decoupled from the backend deployment. This is a feature of our framework that has proved extremely useful in the case of CoCross. It allowed us to deploy the system and start collecting data quickly, while at the same being able to react to users’ feedback. We can iterate on existing features and provide new ones without experiencing service unavailability. Structured data are stored as RDF resources using the OpenLink Virtuoso server, while the SPIN framework is used for data integrity and validation. The API endpoints are implemented using JAX-RS and the resulting Java war is deployed using the WildFly application server.

### 3.3. The CoCross Web Application for Intensivist

Any intensivist in the intensivists group that has access to the medical record of a patient may access all the data recorded in the ICU recording sessions after their automatic synchronization. 

**Figure 3:**
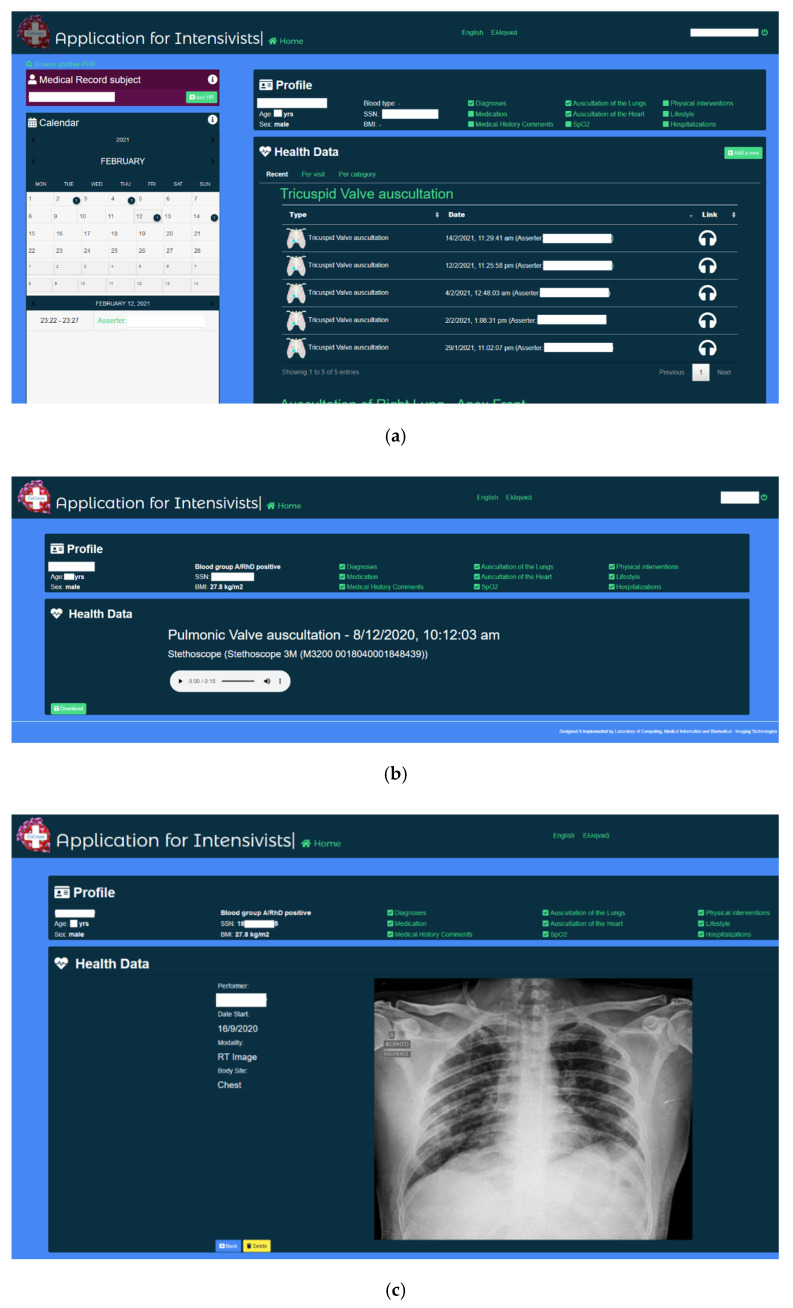
CoCross web application for intensivists (**a**) home screen, (**b**) auscultation playback and (**c**) X-ray review.

Using the CoCross web application, the intensivists manage the contents of each medical record and may perform user registration and record sharing. Along with the presentation, per patient, of all the ICU acquired data, either per data type (e.g., all heart rate measurements) or per recording session, the intensivist may enter numerical, categorical or media files acquired outside of the system. Additionally, ICD10 diagnosis along with basic demographic information (i.e., age, gender) may be entered into the medical record along with DICOM images from imaging modalities (ultrasound, X-ray, etc.). Snapshots from the CoCross web application are shown in Figure 3.

The stored numerical measurements are presented either as tables or as graphs, depending on the number of data points. The intensivists may playback the recorded auscultations via the web browser or download the auscultation locally as a sound file. Similar capability (online view or download) is provided also for DICOM image series. An internal messaging system has been developed to allow exchange of messages among intensivists in specific context. Via this messaging system the intensivist, either inside or outside the ICU, may discuss/comment on a specific patient, recording session or acquisition.

The web application for intensivists is developed using the Laravel framework and it is accessible both via tablet (e.g., inside the ICU) and PC (e.g., outside the ICU). The use of the Laravel framework enhances the maintainability of the application and simplifies the addition of extra features and the collaboration of the development team.

### 3.4. The CoCross Mobile Applications

The CoCross mobile applications manage the interaction with the intensivist and the acquisition of data from the Bluetooth medical devices inside the ICU. The design of the CoCross mobile applications aims to be as simple and unobtrusive as possible for the end-users, while at the same time aiming to be scalable by design allowing the developers to add support for new medical devices. To that end, extending the support of the platform for an additional device, requires only the development of a lightweight application-specific for each device model, managing the communication parameters of the specific model and type of device. These modules have, where possible, no user interface (e.g., oximeter module), this means that acquired measurements are automatically transferred to CoCross4Pros once the measurement procedure is completed or have limited interaction with the user to set up measurement of specific metadata (e.g., the selection of auscultation location of the auscultation module). All module applications communicate with the CoCross4Pros application via the Android inter-process communication (IPC) using the Android interface definition language (AIDL) and they require the existence of the CoCross4Pros application to work properly. Using AIDL the CoCross4Pros defines a set of functions that the modules can call/invoke, to be executed in the context of (i.e., having access to the memory of) the CoCross4Pros application. Doing so, the acquired data are stored linked to the selected patient, and they are, subsequently, synchronized to the specific medical record using the credentials of the responsible intensivist.

**Figure 4:**
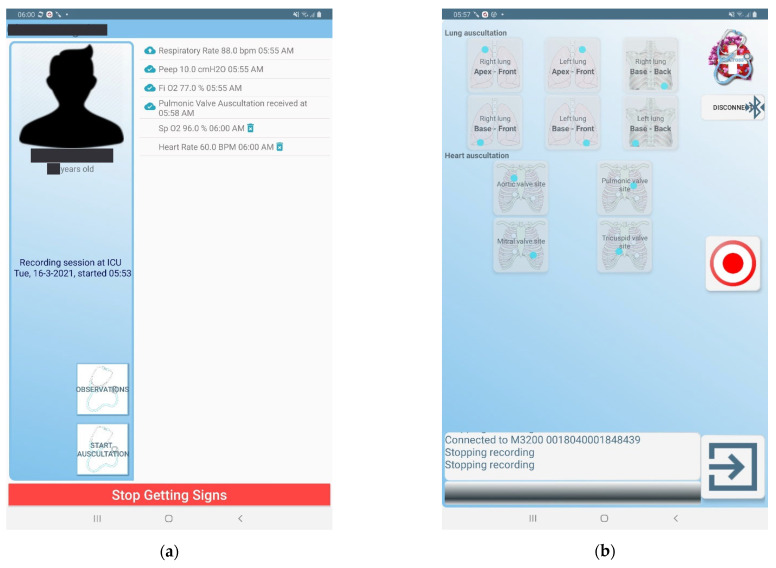
(**a**) CoCross4Pros during an active recording session, (**b**) auscultation module application screen.

The CoCross4Pros application, apart from the definition of the AIDL functions and the implementation of their functionality, also manages (a) the interaction with the user and, (b) the secure transfer of the locally stored data to the CoCross back-end via the data management service. Specifically, it manages the authentication of the user requiring full credentials (email/password) and active network connection at least once. Each user may define a PIN stored only in the tablet to assist faster and, if needed, an off-line login sequence. The intensivist can view a list of patients under his/her responsibility and start a recording session (Figure 4). All data retrieved by the device modules while the recording session is active are linked to the specific recording session and thus to the specific patient.

The CoCross4Pros application also enables the exchange of various notes about the patient or the specific recording session. These notes may include communication between the resident intensivist and the expert/consultant regarding possible questions for the patient or about a specific signal. This functionality is particularly important during the COVID-19 pandemic since non-critical observations may be missed due to the inability to easily transfer objects in and out of the ICU. 

The data of each recording session are stored locally on the tablet once acquired and they are securely transmitted to the CoCross data management service without any user interaction.

**Figure 5:**
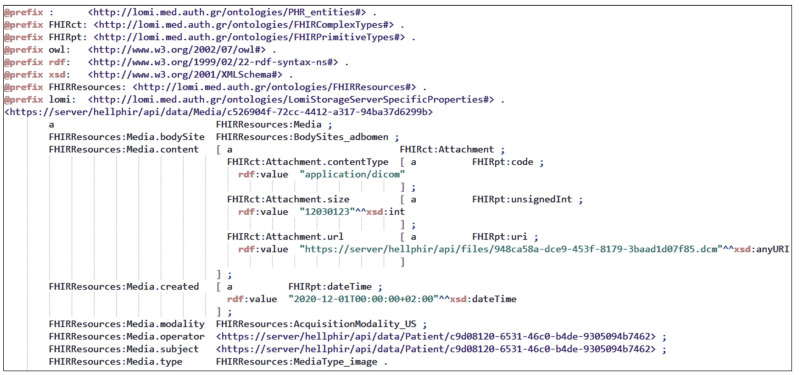
The representation of an uploaded ultrasound DICOM image as FHIR media resource serialized in RDF/turtle.

All data are transmitted and stored at the CoCross back-end as RDF graphs serialized in Turtle (Terse RDF Triple Language). In Figure 5 an example instance of media resource corresponding into an ultrasound DICOM image series and serialized in RDF/turtle is presented. It must be noted that the data stored on CoCross back-end, apart from the patient, they maintain a link to the specific recording session, the responsible intensivist, and the specific acquisition device.

## 4. Results

**Figure 6:**
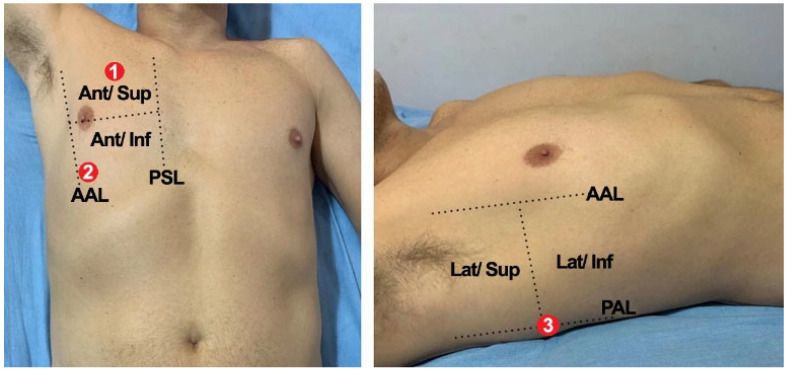
Lung auscultation locations for in ICU patients of CoCross.

The CoCross platform was deployed in June 2020 in two of the COVID-19 reference hospitals in Greece: Initially in the 1st ICU of “G. Papanikolaou” hospital in Thessaloniki (May 2020) and shortly after (June 2020) in the ICU of “Evangelismos” hospital in Athens. Upon permission of the respective scientific boards (ethical approval protocol number 875/20 May 2020 for G. Papanikolaou Hospital study), recordings from patients with COVID-19 receiving care in ICU have been performed and are still ongoing. The protocol included auscultations of the lungs from six locations, namely: the auscultations of the (1) right lung—apex front, (2) right lung—base front, (3) right lung—base back, (4) left lung—apex front, (5) left lung—base front, and (6) left lung—base back, and, from four locations of the heart: the standard auscultations points for the (7) aortic valve, (8) pulmonic valve, (9) mitral valve and, (10) tricuspid valve. In Figure 6 the specific locations for the auscultation of the right lung (i.e., numbers 1–3) of ICU-based patients are presented. Optionally, in each recording session, SpO2 was measured and if feasible lung ultrasound images were captured with a portable device and uploaded as DICOM images.

### 4.1. Deployment Details

In each ICU a 10” tablet, a Bluetooth pulse oximeter (Medisana™ pulse oximeter PM150) and a Bluetooth digital stethoscope (3M™ Littmann^®^ Electronic Stethoscope Model 3200) and a portable ultrasound device (ATL Milano Wireless Ultrasound Probe Convex Color Doppler—C05C) were provided and kept inside the ICU at all times. Since this was the initial deployment, to ensure the feasibility of the study no dedicated expensive servers were deployed. The CoCross backend was deployed in two dedicated and isolated virtual machines (VMs)—one per site. The specific setup offered the required isolation of data among the different clinics/hospitals while also stress-tested the system under real-world conditions. Specifically, since the VMs used were low-end (equivalent to an Intel Core 2 Duo CPU from 2009), the performance of the system was assessed to identify minimum requirements in terms of possible hardware required for new deployments. 

During the first nine months of the deployment of the system, no technical issues were observed resulting in system downtime. No sessions were omitted due to technical issues either in the mobile applications or the CoCross services highlighting a technically robust system. All recorded data were accessible after a few seconds to the web application for intensivists. The specific setup of deployed services adequately supported the limited number of users per site. In detail, each auscultation on a specific location was uploaded from the CoCross4Pros mobile application to the CoCross back-end within approximately 40 s. This period includes a 30 s grace period for aborting the upload if the intensivist wants to discard a recording, for any possible reason (e.g., performed the recording on a different location, or there was external noise during recording). Subsequently, the recordings were available for review via the web application for intensivists. The presentation of the patient dashboard view which includes all the data for the specific patient varies slightly depending on the number of sessions that were performed. For, the current, worst-case scenario, i.e., the patient with the most recording sessions (30 sessions, 175 auscultations, without using cache), the dashboard was presented in 20 s, which was acceptable by the intensivists.

### 4.2. Deployment Statistics

During the first nine months (June 2020–February 2021) of CoCross deployment in the ICUs, 176 patients, 61 females with average age 66.2 ± 12.4 years and 115 males with average age 64.8 ± 11.7 years, were registered to the system with confirmed COVID-19 and at least one auscultation recording session was performed in 134 patients. The patients were monitored from 1 to 46 days with a median value of 5 days, resulting in 508 distinct recording sessions that include 3477 auscultations of 20 s each.

**Figure 7:**
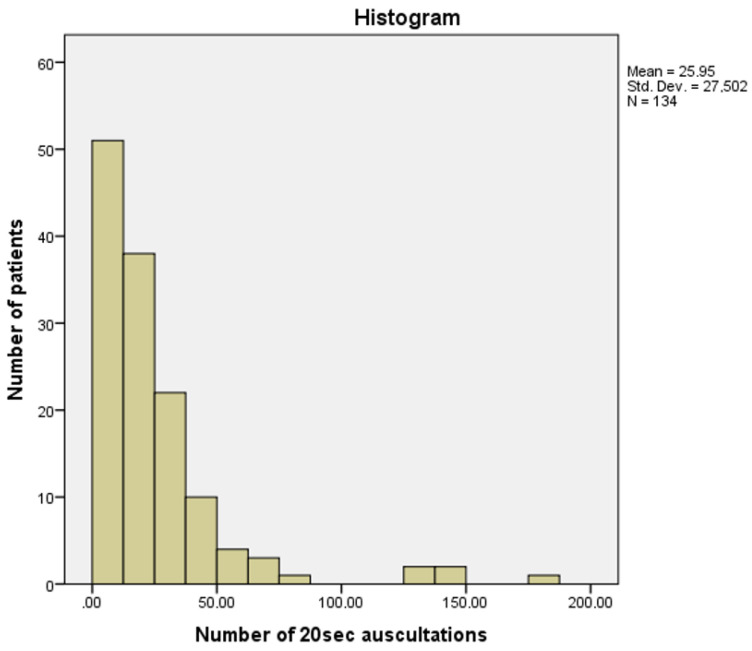
CoCross: histogram of the number of auscultation recordings per patient.

A histogram of the number of auscultation recordings acquired via CoCross per subject is presented in Figure 7.

### 4.3. User Experience

**Figure 8:**
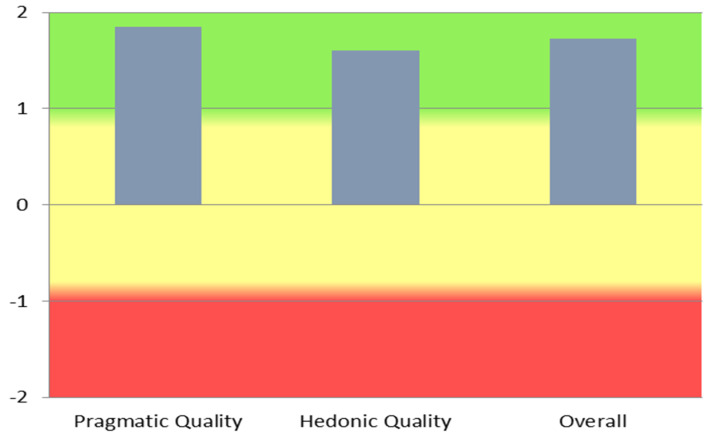
UEQ scales.

It is no longer sufficient to offer applications of new and powerful functionality. Users also expect that they can learn how to use the application without much effort, solve their tasks fast and efficiently and are able to control the interaction at each point. In addition to these goal-oriented interaction qualities, it is also important that the product catches the user’s attention and interest and that using the system is interesting and stimulating. Consequently, hedonic, not directly goal-oriented interaction qualities have to be considered, as well in order to be successful [29]. Although the user base of CoCross, due to its specialization, is small in our deployment, aiming to quantify the user experience regarding the CoCross platform, five out of the seven CoCross users (three intensivists and two pulmonologists), after the first four months of system use, participated in a survey using the short version of the user-experience-questionnaire UEQ-S [30]. In UEQ-S the range of its scale is between −3 (horribly bad) and +3 (extremely good). It must be noted that the survey participants had spent several hours interacting with the system, without any physical technical support due to COVID-19 restrictions. The results are presented in Table 1: detailed per item result of the CoCross UEQ-S survey, and mean values per scale are depicted in Figure 8. The indicative results presented are aligned with the qualitative feedback we received from the users in our discussions. These results, although indicative (due to the small number of users) rate CoCross as an inventive system, that is easy to use and that efficiently addresses the user needs.

## 5. Discussion

Currently the long-term effects of COVID-19 on patients’ health and especially the association of the virus with the development of comorbidities has not been fully examined. Moreover, the optimal management of COVID-19 positive subjects who are asymptomatic or present mild symptomatology at the time of their initial evaluation, mainly patients in higher danger of exhibiting severe forms of the disease (e.g., elderly, persons with potential frailty, with many comorbid conditions or living alone) is still debatable. Social distancing measures imposed on our societies due to the COVID-19 pandemic add an extreme challenge for providing digital personalized point-of-care health tools for achieving prevention in COVID-19 dispersion, early detection, and risk stratification especially in relation to vulnerable groups (for example, patients with chronic cardiorespiratory disease and/or elders).

The problems we are facing with COVID-19 at the moment fall in two categories: (I) identify COVID-19 positives, (II) assess the COVID-19 severity and identify therapeutic interventions.

In the first category, many methods have been developed since last year. Most of them use imaging analytics largely from X-rays since this modality is easy to use widely in clinical and primary health care settings [20,31,32]. Another class of category I approaches uses advanced wearable point of care sensors analyzing coughing sound characteristics for the classification of the subjects [16,33,34]. Other approaches are based on biomolecular analysis (PCR, rapid tests), as well as assessing smell and taste functionality [35], but these techniques are still difficult to apply in larger target groups and their efficiency needs to be shown [36].

In the second category, there is still a long way to go. The severity assessment is difficult to be derived from X-rays, CT or coughing or single biosignals, even from PCR tests. It is our view that the first environment for assessing the COVID severity is either the COVID clinics or the ICU environment. The reason is that patients who enter the ICU have been definitely diagnosed with health-threatening COVID-19 driven lung dysfunction, which in most cases is combined with heart, kidney and other organs and systems impairments.

Our goal in this paper was to present CoCross, a new system used for the creation and development of a dynamic database resulting from multisource/multimodal recordings in the COVID-19 ICUs from two Greek hospitals. These data are aimed to be used for the assessment of the COVID-19 patient status and for the medical decision support in prescribing the necessary interventions for the COVID-19 patients’ treatment and positive ICU outcomes. We believe that systems like CoCross provide necessary and innovative biomedical informatics platforms for the development of COVID-19 annotated ICU databases, which in turn, are expected to trigger methodologies and analytics tools for COVID-19 and at large lung digital diagnostics, as well as better management of multi-morbid patients with lung disease as the primary disease [37].

The accumulated experience from the initial deployment of CoCross inside the ICU on COVID-19 patients has been extremely positive. The system has offered the ICU physicians the unique capability to have a clear view of the auscultation findings of their patients, has helped them identify severe complications at an early stage and guide proper therapeutic interventions accordingly. The storage of the sound files and the ability of a group of intensivists to listen to them was an added value leading to more informed and scientifically robust choices. The evaluation study of the system constitutes one of the first attempts to capture the lung sounds and their variations over time in critically ill COVID-19 patients and combine metrics from X-rays and lung ultrasounds for the more accurate and efficient assessment and diagnosis of COVID-19 patients in the ICU [30]. Future analyses of the spectral traits of the acquired signals could allow for correlations with certain disease complications and alterations in cardiorespiratory dynamics [38,39,40].

One of the great potentials provided by the CoCross system and the resulting database is the possibility to fuse information from the analytics from lung ICU Ultrasound (LUS) [41] with X-ray findings, lung sound characteristics and standard ICU measurements (like measurements of the respiratory mechanics or arterial blood gases analysis) and scores (e.g., SOFA, APACHE II). This is an additional reason that the CoCross database will have a high impact to our scientific community, judging from the success of the ICBHI database [13,25].

The ongoing study using the CoCross platform will address the need for an alternative method of bedside monitoring to patients with COVID-19 treated in the ICU, ideally combining different diagnostic techniques, and transmitting the recordings wirelessly for further assessment by the ICU team of intensivists. This CoCross functionality can address several COVID-19 ICU organizational and management issues related to the optimal resource management of the ventilators, shortage, and safety of the specialized medical staff [42].

The use of the CoCross system is expected to result in the development of a large, annotated database of lung/heart sounds and lung imaging to be leveraged by machine learning and clinical research protocols and advance the Integration Information Initiative (III) including micro-nanosensors, ICT solutions and health care systems management [15].

## 6. Conclusions

In this paper we have presented CoCross, a platform that enables monitoring, recording and fusion of clinical information with chest sounds and imaging of COVID-19 ICU patients. The resulting multisource/multimodal dynamic database is aiming to fuel future research regarding open issues, such as, severity and disease progress prognosis and identification of the optimal treatment. The platform was successfully deployed in two ICUs in Greece and resulted in the acquisition of multisource data from 176 ICU patients, corresponding to an average monitoring period of 5 days, including a dataset with 3477 distinct 20-s auscultations. The platform is well accepted and positively rated by the users, who also exhibited that they could learn how to use the application without much effort and with minimal technical support.

CoCross is easy to deploy, requires minimal hardware cost and has proven to be technically robust. It has demonstrated its capabilities during use in ICU, collecting annotated auscultation sounds of lung and heart, based on commercially certified stethoscope, and providing almost real-time assessment of these sounds by the experts in and outside of the ICU. The system also supports several commercial certified devices for biosignal acquisition and health monitoring, as well as support for uploading/review of DICOM imaging from different modalities.

As limitations of CoCross we identify the current support for only one stethoscope model, and that the tools developed for monitoring the expansion of the generated database are not integrated into the web application for intensivists.

The successful deployment at Papanikolaou and Evangelismos hospitals led to the adoption of the system by additional ICUs and pneumology departments. A CoCross clone was deployed in March 2021 for the ICU of the university hospital of Alexandroupolis, Greece, later that year on the pneumology department of the CHUC—Centro Hospitalar e Universitário de Coimbra (University Hospital of Coimbra) and, on December 2021 CoCross was deployed in the ICU of AHEPA university hospital in Thessaloniki Greece. In parallel with the straightforward task of setting up a system clone, additional concepts to be managed were required by local experts such as additional ventilator parameters, recording of interventional events such as intubation or extubation, etc. The new concepts were added, with minimal effort, to the local ontology and to the application for intensivists. Changes applied by the new requirements were propagated to the existing deployments enhancing the system without altering either the existing data or their semantics.

Two are the main pillars related to future efforts linked with CoCross apart from network expansion. The first concerns the aggregation of the data and the preliminary analysis of the data that CoCross is acquiring to assess the quality/completeness of the generated database, and the second is the adoption of new devices for auscultation that provide a better quality of acquired signals.

## Figures and Tables

**Table 1:** Detailed per item result of the CoCross UEQ-S survey.

Item	Scale (Quality)	Mean (Std. Dev)	Negative	Positive
1	Pragmatic	1.4 (±1.1)	obstructive	supportive
2		1.6 (±0.9)	complicated	easy
3		2.6 (±0.5)	inefficient	efficient
4		1.8 (±1.1)	confusing	Clear
	Pragmatic quality mean: 1.85 (±0.74)			
5	Hedonic	0.8 (±1.9)	boring	exciting
6		1.6 (±2.2)	not interesting	interesting
7		2.0 (±1.0)	conventional	inventive
8		2.0 (±1.0)	usual	leading edge
	Hedonic quality mean: 1.60 (±0.95)			
	Overall UEQ-S score: 1.725 (±0.46)			

## Data Availability

Not applicable.
